# *Dracocephalum moldavica* Ethanol Extract Suppresses LPS-Induced Inflammatory Responses through Inhibition of the JNK/ERK/NF-κB Signaling Pathway and IL-6 Production in RAW 264.7 Macrophages and in Endotoxic-Treated Mice

**DOI:** 10.3390/nu13124501

**Published:** 2021-12-16

**Authors:** Kyeong-Min Kim, So-Yeon Kim, Tamanna Jahan Mony, Ho Jung Bae, Sang-Deok Han, Eun-Seok Lee, Seung-Hyuk Choi, Sun Hee Hong, Sang-Deok Lee, Se Jin Park

**Affiliations:** 1 Department of Food Biotechnology and Environmental Science, School of Natural Resources and Environmental Sciences, Kangwon National University, Chuncheon 24341, Korea; kasbai@kangwon.ac.kr (K.-M.K.); ykims95@kangwon.ac.kr (S.-Y.K.); 202016097@kangwon.ac.kr (S.-D.H.); dmstjr0806@kangwon.ac.kr (E.-S.L.); chltmdgur96@kangwon.ac.kr (S.-H.C.); 2 Agriculture and Life Science Research Institute, Kangwon National University, Chuncheon 24341, Korea; tjmonycvasu@gmail.com (T.J.M.); baehj321@kangwon.ac.kr (H.J.B.); 3 School of Applied Science in Natural Resources & Environment, Hankyong National University, Anseong 17579, Korea; shhong@hknu.ac.kr; 4 Division of Forest Science, Kangwon National University, Chuncheon 24341, Korea

**Keywords:** lipopolysaccharide, inflammation, interleukin-6, sepsis, *Dracocephalum moldavica*

## Abstract

The excessive synthesis of interleukin-6 (IL-6) is related to cytokine storm in COVID-19 patients. Moreover, blocking IL-6 has been suggested as a treatment strategy for inflammatory diseases such as sepsis. Sepsis is a severe systemic inflammatory response syndrome with high mortality. In the present study, we investigated the anti-inflammatory and anti-septic effects and the underlying mechanisms of *Dracocephalum moldavica* ethanol extract (DMEE) on lipopolysaccharide (LPS)-induced inflammatory stimulation in RAW 264.7 macrophages along with septic mouse models. We found that DMEE suppressed the release of inflammatory mediators NO and PGE_2_ and inhibited both the mRNA and protein expression levels of iNOS and COX-2, respectively. In addition, DMEE reduced the release of proinflammatory cytokines, mainly IL-6 and IL-1β, in RAW 264.7 cells by inhibiting the phosphorylation of JNK, ERK and p65. Furthermore, treatment with DMEE increased the survival rate and decreased the level of IL-6 in plasma in LPS-induced septic shock mice. Our findings suggest that DMEE elicits an anti-inflammatory effect in LPS-stimulated RAW 264.7 macrophages and an anti-septic effect on septic mouse model through the inhibition of the ERK/JNK/NF-κB signaling cascades and production of IL-6.

## 1. Introduction

The inflammatory reaction (inflammation) is the foremost defense mechanism with a complex biological response from the body’s tissues, after invasion of harmful stimuli. The inflammatory response is a multifactorial function accompanied by the activation of a signaling pathway that regulates inflammatory mediator levels in the host tissues [1]. The biological processes of the immune system are triggered by several factors, including pathogens, irritants, damaged cells and endotoxins such as lipopolysaccharide (LPS). LPS is the major glycoprotein that constitutes and acts as a highly immunogenic and the most important component of the outer cell wall of Gram-negative bacteria [2]. LPS binds with the binding protein (LBP) and is recognized by Toll-like receptors—most commonly by TLR4, which activates the immune system as part of the innate immune response [3]. The activated TLR4 complex signaling pathway, that includes the myeloid differentiation primary-response protein 88 (MyD88)-dependent pathway, can stimulate the upregulation of inflammatory gene expression [4]. The amino terminus of MyD88 recruits the IL-1 receptor-associated kinase (IRAK) family and then the phosphorylated MyD88/IRAK complex binds to TNF receptor-associated factor 6 (TRAF6) [5]. The transforming growth factor B-activated kinase (TAK1) complex is activated by TRAF6 and phosphorylated NF-κB, which translocate to the nucleus to initiate transcription and activate the gene expression of cytokines [6]. At the same time, TAK1 activates the MAPK family members, including JNK1/2, ERK1/2 and p38, which enter the nucleus and activate activator protein 1 (AP-1) [7].

Activated AP-1 and NF-κB signaling results in the secretion of proinflammatory cytokines, such as tumor necrosis factor-α (TNF-α), interleukin-6 (IL-6) and interleukin-1β (IL-1β), and chemokines. Excessive production of inflammatory cytokines and chemokines, which has been designated as a cytokine storm, leads to systemic inflammatory response syndromes such as COVID-19, rheumatoid arthritis and sepsis [8,9,10]. Sepsis is defined as a life-threatening organ dysfunction caused by the dysregulated host response to invading pathogens [11]. It is one of the typical diseases in systemic inflammatory response syndrome caused by a cytokine storm [12]. The main problem of sepsis is the high mortality due to the absence of distinct therapeutic strategies for increasing the survival rate [13,14]. The early administration of antibiotics is associated with decreased mortality, but patients who receive antibiotics such as vancomycin and piperacillin/tazobactam may have harmful outcomes, such as acute kidney injury [15,16]. Therefore, it is necessary to develop safe anti-septic therapies that positively increase the survival rate. Importantly, a number of patients with septic shock have increased IL-6 in their plasma levels, according to the ICU database [17]. Moreover, it has been reported that patients who had reduced IL-6 levels were 5.68 times more likely to survive than non-survivors [18]. Thus, blocking IL-6 levels may be an effective therapeutic strategy for reducing cytokine storm diseases.

**Figure 1:**
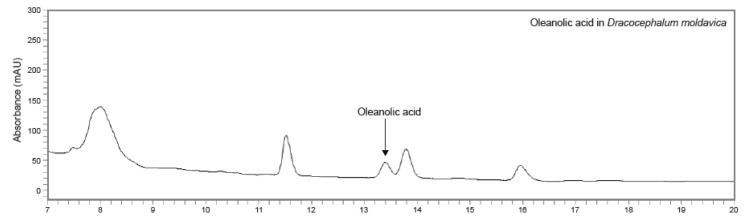
HPLC-UV chromatogram analysis of oleanolic acid in *D. moldavica* with detector responses at 210 nm.

Moldavian balm (*Dracocephalum moldavica*), known as Yixin Badi Ran Gibuya, is an annual herb that belongs to the family Lamiaceae [19]. *D. moldavica* is native in northern China, southeastern Xinjiang and eastern Europe. Furthermore, this herb has been used in traditional Uygur herbal drugs for headache, liver disorders and cardiovascular diseases, such as coronary heart disease, for many centuries [20]. This herbaceous plant has been reported to contain active components with anti-inflammatory and antioxidant effects, such as hydroxycinnamic acids, flavonoids and rosmarinic acid [21,22]. Rosmarinic acid (RA) has also been reported to significantly inhibit lung cell apoptosis and decrease the level of p53 in LPS-induced septic mice by inhibiting the activation of the GRP78/IRE1alpha/JNK pathway [23]. Additionally, RA has shown to downregulate the levels of TNF-a, IL-6 and HMGB-1 in LPS-induced RAW 264.7 cells by inhibiting the IkB kinase pathway [24]. Moreover, we confirmed that oleanolic acid (OA) was detected in *D. moldavica* ethanolic extract (DMEE), with an average level of approximately 4.32 ± 0.02 mg/g (Figure 1). OA has been reported to be useful as a therapeutic strategy for vascular inflammatory diseases by inhibiting hypermeability, the expression of cell adhesion molecules (CAMs) and the migration of leukocytes [25]. In addition, OA can regulate apoptosis and inflammation in spinal cord injury by blocking p38 and JNK [26].

Based on the anti-inflammatory effects of *D. moldavica* [27,28] and its ingredients, we speculated that DMEE may attenuate LPS-induced inflammatory responses and septic shocks in mice. In this study, we studied the anti-inflammatory effect and underlying mechanisms of DMEE on LPS-stimulated RAW 264.7 macrophages. Additionally, we further investigated the anti-septic effect of DMEE in LPS-induced septic shock mice.

## 2. Materials and Methods

### 2.1. Animals

Five-week-old male mice were supplied by Orient Bio (Seongnam, Korea). In each group, twelve mice were housed per cage with an optimized temperature of 21–25 °C and a 12 h light–dark cycle. Animals were provided with ad libitum food and water throughout the experimental period. All the animal experiments were conducted by following the ethical guidelines of the Institutional Animal Care and Use Committee of Kangwon National University (KW-200128-1).

### 2.2. Preparation of an Ethanolic Extract of D. moldavica

Dried leaves of *D. moldavica* were collected by Professor Xiang-Qian Liu (School of Pharmacy, Hunan University of Chinese Medicine, Changsha, China). We previously reported the preparation method of ethanolic extract of *D. moldavica* (DMEE) [29]. Briefly, dried *D. moldavica* leaves were mixed with 70% ethanol to extract DMEE, twice for 2 h by using an ultrasonic bath. After extracting, it was filtered and consequently concentrated in a water bath under vacuum pressure. Afterward, frozen and lyophilized phases were obtained. The obtained extract was stored at −20 °C until use.

### 2.3. High-Performance Liquid Chromatography (HPLC) Analysis

The HPLC analysis was performed to determine the levels of oleanolic acid in *D. moldavica* with a Perkin Elmer Flexar QUATERNARY Pump (PerkinElmer, Inc., Shelton, CT, USA) and a PDA LC detector (PerkinElmer, Inc., Shelton, CT, USA). The samples were separated by a YMC Pack-Pro C18 column (25 cm × 4.6 mm) in gradient elution mode. Two mobile phases were obtained that comprised 0.2% acetic acid in H_2_O (A) and acetonitrile (B); the overall flow rate was 0.8 mL/min. The column temperature was 30 °C and the injection volume was 5 μL. The gradient conditions of oleanolic acid were 10% (A) and 90% (B) for 0–45 min. The test solution (*D. moldavica*) was weighed (60 mg) and dissolved at 20 mg/mL in MeOH. Then, the solution was sonicated for 30 min and filtered using a 0.45 μm PVDF membrane filter. Similarly, the standard solution (oleanolic acid) was weighed (1 mg) and dissolved at a concentration of 1 mg/mL in MeOH. The standard solution was sonicated and filtered under the same conditions as the test solution. The analysis of oleanolic acid in DMEE was detected at a 210 nm wavelength. The oleanolic acid composites were calculated by applying the following calibration curve equation: oleanolic acid, y = 2567.7x + 23708, R^2^ = 1. The average level of oleanolic acid in *D. moldavica* was approximately 4.32 ± 0.02 mg/g (Figure 1).

### 2.4. Materials

Tetrazolium bromide (MTT), Dimethyl sulfoxide (DMSO), LPS, 3-(4,5-dimethylthiazol-2-yl)-2,5-diphenyl, Griess reagent and sodium nitrite were purchased from Sigma Chemical Co. (St. Louis, MO, USA). Penicillin–streptomycin (P/S), Dulbecco’s phosphate buffered saline (DPBS), Dulbecco’s modified Eagle’s medium (DMEM) and DEPC water were purchased from Welgene (Gyeongsan, Korea). Fetal bovine serum (FBS) was provided by Atlas Biologicals (Fort Collins, CO, USA). RNAiso Plus was purchased from Takara Bio Inc. (Kusatsu, Japan). Chloroform, 2-propyl alcohol, acetone and olive oil were purchased from Daejung (Seongnam, Korea). Primers for cyclooxygenase-2 (COX-2), inducible nitric oxide synthase (iNOS), TNF-α, IL-1β, IL-6 and β-actin oligonucleotide were purchased from Integrated DNA Technologies (Coralville, IA, USA). We used enzyme-linked immunosorbent assay (ELISA) kits for prostagladin E_2_ (PGE_2_) from R&D Systems (Minneapolis, MN, USA) and IL-6 and IL-1β from Abcam (Cambridge, UK). *TransScript*^®^ All-in-One First-Strand cDNA Synthesis SuperMix for qPCR (One-Step gDNA Removal) was obtained from TransGen Biotech Co. (Beijing, China). PowerSYBR^®^ Green PCR Master Mix from Applied Biosystems was purchased from Thermo Fisher Scientific (Rockford, IL, USA). Antibodies against p38, JNK, ERK, p65, phosphorylated p38 (p-p38), phosphorylated JNK (p-JNK), phosphorylated ERK (p-ERK) and phosphorylated p65 (p-p65) were supplied by Cell Signaling Technology, Inc. (Danvers, MA, USA). Other materials were purchased from usual commercial sources and ensured the highest available grade.

### 2.5. Cell Culture

Raw 264.7 cells—mouse-originated macrophages (RAW 264.7)—were supplied by the Korean Cell Line Bank (KCLB, Seoul, Korea). For cell culture, DMEM (100 units/Ml), P/S and 10% FBS were used as media. The cultured cells were incubated at 37 °C and 5% CO_2_ and subsequently subcultured every two days.

### 2.6. Analysis of Cell Viability

The MTT assay was performed to measure the cell viability. First, DMEE-treated cells were incubated for 24 h; subsequently, the MTT assay was conducted. After incubation with MTT solution (5 mg/mL), the cells were mixed with PBS and incubated at 37 °C for 4 h. After that, the MTT solution was removed and the produced purple formazan crystals were solubilized in DMSO (100 μL/well) as described by [30]. The optical density was measured at 540 nm with a microplate spectrophotometer (SpectraMax, Molecular Devices, Sunnyvale, CA, USA).

### 2.7. Determination of Nitric Oxide Production

RAW 264.7 cells were pretreated with different concentrations of DMEE (50, 100, 200 and 400 μg/mL) for 1 h; later, they were stimulated with LPS at a concentration of 1 μg/mL for 24 h. Nitrite accumulation in the culture medium was considered as an indicator of nitric oxide (NO) production. The total NO production was measured with Griess reagent. Equal volumes of 100 µL of the supernatant and Griess reagent were mixed for 10 min [31]. The optical density was determined using a microplate reader (SpectraMax, molecular Devices, Sunnyvale, CA, USA) at 540 nm. The total of nitrite in the samples was determined based on a sodium nitrite standard curve.

### 2.8. PGE_2_, IL-6 and IL-1β Assays

The expression levels of PGE_2_, IL-6 and IL-1β, both in macrophage culture medium and plasma, were measured using commercial ELISA kits. The cells were pretreated with DMEE at various concentrations (from 50 μg/mL to 400 μg/mL) for 1 h; afterwards, they were treated with LPS (1 μg/mL) for 24 h. Cytokine expression in the cell was determined by ELISA, referring to the manufacturer’s given protocol.

### 2.9. RNA Extraction and Real Time-Quantative PCR (RT-PCR)

The mRNA expressions of iNOS, COX-2, IL-6 and IL-1β were detected by performing RT-PCR. Total RNA was extracted using RNAiso PLUS (Takara, Otsu, Japan). From 1 μg of total RNA, cDNA was synthesized using the All-in-One FirstStrand cDNA Synthesis SuperMix, as previously described by [32]. The synthesized cDNAs were used as template for qRT-PCR using a QuantStudio 3 (Applied Biosystems, Foster City, CA, USA) system with POWER SYBR Green PCR master mix and gene-specific primers (Table 1). A dissociation curve analysis of iNOS, COX-2, IL-6, IL-1β and β-actin demonstrated a single peak. The expression levels of target genes were quantified by duplicate measurements and normalized with the 2^−ΔΔCT^ method relative to control β-actin. The PCR analyses were performed under the following conditions: 40 cycles of 95 °C for 15 s; 57 °C for 20 s; and 72 °C for 40 s.

### 2.10. Western Blot Analysis

For immunoblot analysis, LPS-stimulated cells were washed twice with ice-cold PBS. Total proteins were isolated from the cells using a lysis buffer with cocktails of protein inhibitors and then harvested with a cell scraper [33]. Total cellular protein was quantified using the Bradford assay. The protein (20 μg/well protein) was loaded to 10% SDS-PAGE and then transferred to PVDF membranes [34]. The membranes were blocked with 5% skimmed milk for 2 h and then incubated with primary antibodies against p-JNK (Cell Signaling Technology (Danvers, MA, USA), 1:1000), p-ERK (Cell Signaling Technology, 1:1000), p-p65 (Cell Signaling Technology, 1:1000), JNK (Cell Signaling Technology, 1:1000), ERK (Cell Signaling Technology, 1:1000), p65 (Cell Signaling Technology, 1:1000), iNOS (Cell Signaling Technology, 1:500), COX-2 (Cell Signaling Technology, 1:1000), or β-actin (Cell Signaling Technology, 1:500) at 4 °C overnight. After washing, the membranes were again incubated for 2 h at room temperature with a secondary antibody (Cell signaling, 1:1000). The probed membranes were developed with enhanced chemiluminescence. The immunoblots were imaged using an LAS-500 mini-imager (General Electric, Boston, MA, USA) and analyzed with the ImageJ program. The phosphorylation level was determined by calculating the ratio of phosphorylated protein to the total protein on the same membrane; this was measured to determine the level of phosphorylation.

### 2.11. LPS-Induced Septic Shock Mice

To examine the effect of DMEE on LPS-induced modality changes in septic shock mice (*n* = 12 per group), the mice were orally treated with DMEE (50, 100 and 200 mg/kg body weight) or vehicle (0.9% saline) for 7 days. To induce septic shock in the mouse models, LPS at 25 mg/kg was injected intraperitoneally to mice and the survival of the mice was monitored for 3 days. In the satellite study (*n* = 4 per group), the mice were sacrificed at 12 h after LPS injection and a blood sample was collected to determine proinflammatory cytokine levels in the plasma.

### 2.12. Statistical Analyses

The statistical analyses were performed using GraphPad Prism Version 8.0 (GraphPad, La Jolla, CA, USA). All data are expressed as the mean ± S.E.M. The data were analyzed by a one-way analysis of variance (ANOVA), followed by a Student–Newman–Keuls test for multiple comparisons. A *p* < 0.05 was considered as a significant statistical value.

## 3. Results

### 3.1. HPLC-UV Chromatograms Analysis of Oleanolic Acid in DMEE

Based on the reported anti-inflammatory property of oleanolic acid, we performed the HPLC-UV detector analysis of oleanolic acid in DMEE. We confirmed that the average level of oleanolic acid in DMEE was approximately 4.32 ± 0.02 mg/g (Figure 1).

### 3.2. DMEE Inhibits the LPS-Stimulated Production of Inflammatory Mediators and iNOS and COX-2 Expression in RAW 264.7 Cells

**Figure 2:**
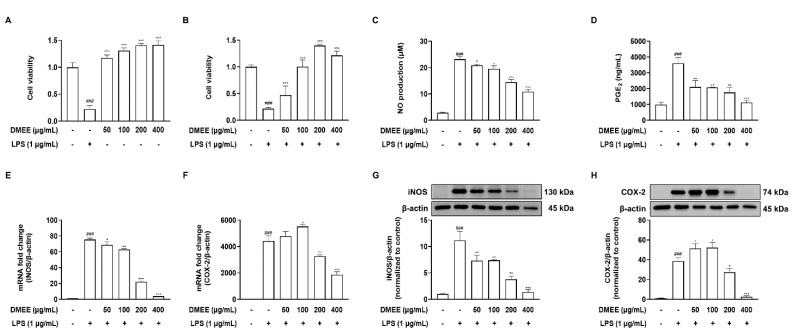
Effects of *D. moldavica* on LPS-induced inflammatory response in RAW 264.7 cells. Cells were pretreated with *D. moldavica* for 1 h and then treated with LPS (1 μg/mL) for 24 h. Cell viability was determined by MTT assay (*n* = 5) (**A**,**B**). The production of NO was measured by Griess reaction (*n* = 3) (**C**). The level of PGE_2_ was measured by PGE_2_ ELISA kit (*n* = 3) (**D**). The mRNA expression of iNOS and COX-2 was determined by RT-PCR (*n* = 3) (**E**,**F**). The protein levels of iNOS and COX-2 were measured by Western blotting and the quantification of iNOS and COX-2 was normalized to the control (*n* = 3) (**G**,**H**). The data shown are representative of three independent experiments and indicate mean ± S.E.M. *^###^ p* < 0.001 versus the vehicle-treated controls; * p < 0.05, ** p < 0.01 and *** p < 0.001 versus the LPS-treated group.

We conducted an MTT assay to exhibit the effect of DMEE on the viability of RAW 264.7 macrophages. No cytotoxicity of DMEE was observed at any tested concentration (50, 100, 200 and 400 μg/mL) (Figure 2A). Moreover, DMEE blocked cell death stimulated with LPS at concentrations above 100 μg/mL (Figure 2B). To determine the effect of DMEE on LPS-stimulated NO and PGE_2_ production, the cells were pretreated with DMEE for 1 h and then treated with LPS (1 μg/mL) for 24 h. We observed that DMEE suppressed the LPS-induced NO and PGE_2_ production in a dose-dependent manner (Figure 2C,D). Furthermore, we found that the mRNA expression of iNOS and COX-2, which synthesize NO and PGE_2_, was significantly reduced at the DMEE concentrations of 200 and 400 μg/mL (Figure 2E,F). We also observed that the protein expression of iNOS and COX-2 were decreased by DMEE treatment in RAW 264.7 cells (Figure 2G,H). These results indicate that DMEE moderates the production of proinflammatory mediators NO and PGE_2_ via the suppression of iNOS and COX-2 expression.

### 3.3. DMEE Reduced the Secretion of Proinflammatory Cytokines in LPS-Stimulated RAW 264.7 Cells

**Figure 3:**
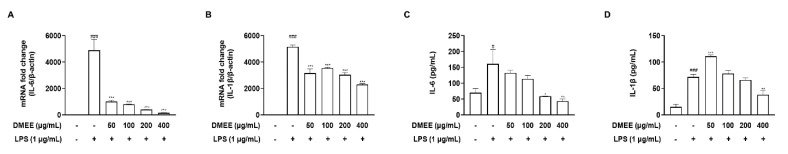
Effects of *D. moldavica* on LPS-induced proinflammatory cytokine expression in RAW 264.7 cells. Cells were pretreated with *D. moldavica* for 1 h and then treated with LPS (1 μg/mL) for 24 h. The mRNA expression of IL-6 and IL-1β was determined by RT-PCR (*n* = 3) (**A**,**B**) and the secretion of IL-6 and IL-1β was measured by IL-6 and IL-1β ELISA kit (*n* = 3) (**C**,**D**). The data shown are representative of three independent experiments and indicate mean ± S.E.M. *^#^* *p* < 0.05, *^###^ p* < 0.001 versus the vehicle-treated controls; * p < 0.05, ** p < 0.01 and *** p < 0.001 versus the LPS-treated group.

During inflammatory responses, immune cells such as macrophages secrete the proinflammatory cytokines that induce various inflammation reactions and the production of NO and PGE_2_ [35]. Thus, inflammatory cytokines are pleiotropic molecules that play a pivotal role in inflammatory reactions [36]. For that reason, we investigated the inhibitory effects of DMEE on the release of proinflammatory cytokines such as IL-6 and IL-1β in RAW 264.7 cells. Macrophages were pretreated with DMEE for 1 h and consequently stimulated with LPS (1 μg/mL) for 24 h. The mRNA expression level of proinflammatory cytokines was determined in the collected medium by RT-PCR analysis. As shown in Figure 3A,B, the mRNA expression levels of IL-6 and IL-1β were significantly increased in LPS-stimulated macrophages compared to the control, which were dose-dependently inhibited by DMEE. We further determined whether treatment with DMEE downregulated the protein levels of IL-6 and IL-1β by using ELISA. Similar to the mRNA expression, the protein expression of IL-6 and IL-1β was also significantly reduced at 400 μg/mL DMEE (Figure 3C,D).

### 3.4. DMEE Suppresses the MAPK/NF-κB Pathway

**Figure 4:**
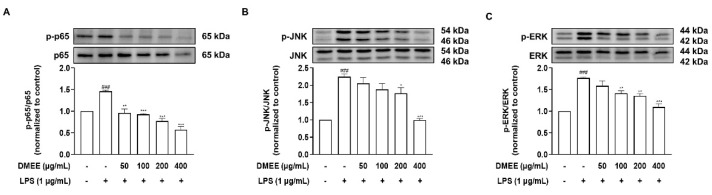
Effects of *D. moldavica* on the MAPK/NF-κB pathway in RAW 264.7 cells. Cells were pretreated with *D. moldavica* for 1 h and then treated with LPS (1 μg/mL) for 30 min. The phosphorylation activity was normalized to the untreated control group. The expression of phospho-JNK, JNK, phospho-ERK, ERK, phosphor-p65, p65 and β-actin was determined by Western blotting (*n* = 3) (**A**–**C**). The data shown are representative of three independent experiments and indicate mean ± S.E.M. *^###^* *p* < 0.001 versus the vehicle-treated controls; * p < 0.05, ** p < 0.01 and *** p < 0.001 versus the LPS-treated group.

After LPS binds to TLR4, it activates the phosphorylation of transcription inducers, for example the MAPK and NF-κB pathways. It is known that the expression of inflammatory cytokines in LPS-induced RAW 264.7 macrophages is associated with the MAPK/NF-κB phosphorylation pathway [37]. Therefore, we investigated whether DMEE inhibited the phosphorylation of p65, a subunit of NF-κB. The phosphorylation of p65 was increased by LPS treatment, but DMEE significantly reduced LPS-stimulated phosphorylation of p65 in RAW 264.7 cells (Figure 4A). Next, we wanted to identify which kinase was involved in the regulation of NF-κB activity. Our results demonstrate that the phosphorylation of JNK 1/2 and ERK 1/2 was increased by LPS stimulation, but DMEE inhibited the phosphorylation of JNK 1/2 and ERK 1/2 (Figure 4B,C). These results indicate that the anti-inflammatory effects of DMEE may be associated with its inhibitory properties on JNK- and ERK-mediated NF-κB activation.

### 3.5. DMEE Enhances the Survival Rate and Reduces the Level of IL-6 in Plasma in LPS-Stimulated Septic Shock in Mice

**Figure 5:**
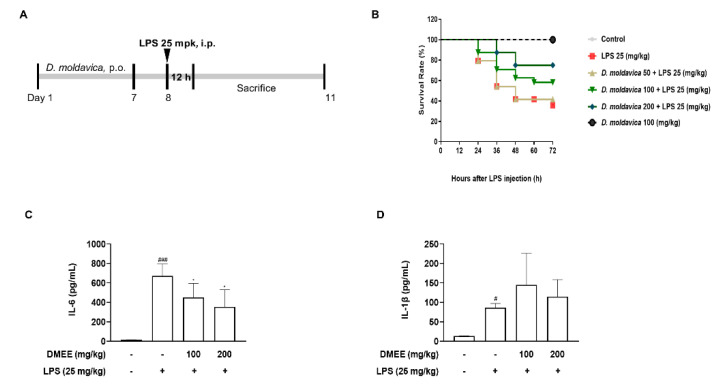
Effect of *D. moldavica* on the survival rate and level of IL-6 in plasma in LPS-induced septic shock mice. Mice were administered with *D. moldavica* (50, 100 and 200 mg/kg p.o.) or vehicle (0.9% saline) for 7 days and then injected with LPS (25 mg/kg, i.p.). The survival rate was measured for 3 days and blood samples were collected 12 h after LPS injection. Timetable of the LPS-induced septic shock mouse model (**A**). Survival rate of the group injected with *D. moldavica* or LPS (control *D. moldavica* 100 mpk, 100%; LPS 25 mpk, 38%; *D. moldavica* 50 mpk + LPS, 42%; *D. moldavica* 100 mpk + LPS, 58%; *D. moldavica* 200 mpk + LPS, 75%) (*n* = 8/group) (**B**). The levels of IL-6 and IL-1β in plasma were determined by ELISA kit (*n* = 4/group) (**C**,**D**). The data shown are representative of three independent experiments and indicate mean ± S.E.M. *^#^* *p* < 0.05 and *^###^* *p* < 0.001 versus the vehicle-treated controls; * p < 0.05 versus the LPS-treated group.

Based on the anti-inflammatory activity of DMEE in vitro, we examined the anti-septic effects of DMEE on LPS-induced septic shock mice (Figure 5A). We orally treated mice with DMEE at the different concentrations of 50, 100 and 200 mg/kg for seven days and then septic shock was induced by LPS (25 mg/kg, i.p.). At 3 days after LPS injection, the survival rate of the LPS-only injected group was the lowest (38%) and the survival rates of the septic shock mice in the group treated with DMEE increased dose-dependently (from 42% to 75%) (Figure 5B). At 12 h after LPS injection, blood samples were collected to measure proinflammatory cytokines, such as IL-6 and IL-1β, in the plasma (Figure 5A). The level of IL-6 in plasma was significantly reduced, but the level of IL-1β was unchanged in LPS-induced septic shock mice (Figure 5C,D). These results indicate that DMEE attenuates LPS-induced septic shock in mice via inhibition of IL-6 production.

## 4. Discussion

Sepsis is considered as a systemic inflammatory response syndrome triggered by an immoderate cytokine expression to counter infections [38]. When the septic response is activated by augmented levels of inflammatory mediators and cytokines, such as NO, TNF-α, IL-1β, COX-2 and IL-6, this causes septic shock in terms of tissue damage and multisystem organ dysfunction, which leads to death [39,40]. Although the analysis of septic shock with cytokine storm with the target treatment of TNF and IL-1 showed promising results in reducing morbidity and mortality in septic shock models, no beneficial results were found in clinical trials [41]. Because of the complex biological responses, there are no effective therapeutic strategies for septic shock. Thus, it is important to impede the excessive expression of inflammatory mediators and cytokines known as cytokine storms [42]. Previous studies reported that IL-6 was determined to be a promising target molecule for systemic inflammatory response syndromes such as sepsis. Moreover, IL-6R antagonists may provide improved results for patients with infectious diseases such as COVID-19 or sepsis [43]. Therefore, blockade of IL-6 suggests that it can regulate cytokine storm diseases [44]. In this study, we observed that the administration of *D. moldavica* increased the survival rate up to 75% in LPS-triggered septic shock mice by inhibiting the secretion level of IL-6 in plasma. Therefore, we suggest that *D. moldavica* would be a promising preventive option for cytokine storm in sepsis or COVID-19.

Moldavian balm is an herb plant (*Dracocephalum moldavica*) of the Lamiaceae family used for traditional Uygur medicine. Uygur people have used *Dracocephalum moldavica* as a therapy for cardiovascular diseases such as myocardial ischemia, hypertension and coronary heart diseases [45]. Moreover, total flavonoids isolated from *D. moldavica* have been demonstrated to inhibit the proliferation and migration of intercellular adhesion molecule-1 and vascular cell adhesion molecule-1 in vascular smooth muscle cells by inhibiting NF-κB expression [46]. In addition, *D. moldavica* has been reported to markedly improve on rat cerebral ischemia reperfusion injury by reducing the levels of IL-6, IL-8 and TNF-α and elevating the activities of superoxide dismutase (SOD) and glutathione peroxidase (GSH-Px) [47]. Notably, we found that the DMEE used in this study contained the active component oleanolic acid. Previous studies reported that oleanolic acid exerts anti-inflammatory effects through inhibited the phosphorylation of ERK1/2, p38, JNK1/2 and p65 in LPS-stimulated RAW 264.7 cells [48,49,50]. Other studies also reported that DMEE contains rosmarinic acid and chlorogenic acid that inhibit the inflammatory mediators and inflammatory symptoms in LPS-stimulated RAW 264.7 cells [51,52]. Altogether, we speculate that the terpenoids and phenolic acids contained in DMEE collectively exhibit anti-inflammatory effects.

**Figure 6:**
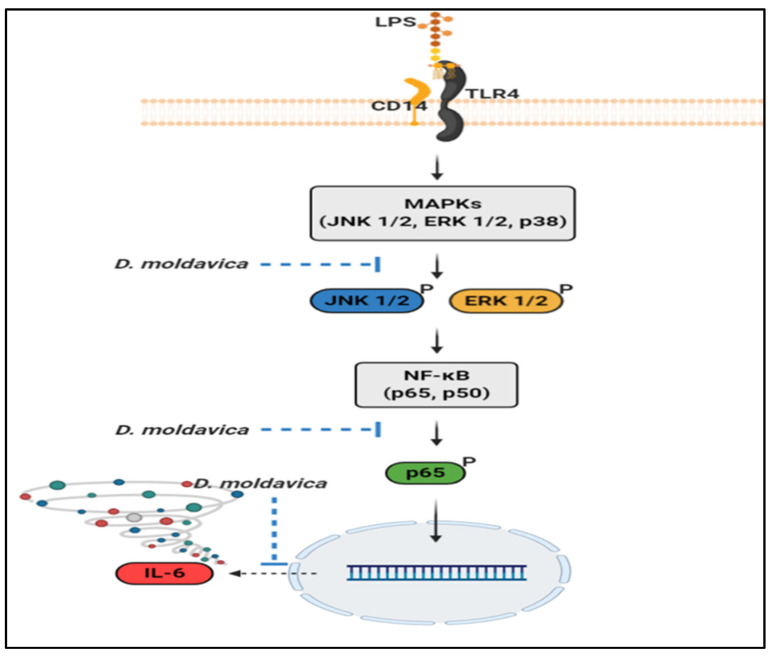
The anti-inflammatory pathways of *D. moldavica* in LPS-stimulated RAW 264.7 macrophages.

NO and PGE_2_ production, which plays key roles in the modulation of immune responses, mediates inflammatory responses, including pain and hypersensitivity [53,54]. Our experimental data showed that DMEE significantly inhibited the production and accumulation of the inflammatory mediators NO and PGE_2_ by decreasing the expression of iNOS and COX-2. We also found that DMEE was related to ameliorating nociception in the formalin test (data not shown). Moreover, DMEE significantly downregulated the phosphorylation of JNK1/2, ERK1/2 and p65 (Figure 6). However, DMEE did not reduce the phosphorylation of p38 in LPS-induced RAW 264.7 cells, since oleanolic acid may facilitate the phosphorylation of p38 [55]. As a result, DMEE reduced the expression of IL-6 and IL-1β in LPS-triggered RAW 264.7 cells by inhibiting the activation of the JNK, ERK and NF-κB pathways. Although the expression of IL-1β in LPS-stimulated RAW 264.7 cells was reduced, it could not be reduced in LPS-induced septic mice. Therefore, we suggest that further research to verify the changes on various inflammatory cytokines in septic mice by DMEE is needed. In addition, we observed that DMEE protected against LPS-induced cell death in RAW 264.7 cells (Figure 2B). However, we cannot rule out that DMEE may enhance cell proliferation but has no protective effects. Therefore, in a future study, we will examine whether the effect of DMEE on LPS-induced cell death is due to its protection or cell proliferation.

In conclusion, our data demonstrated that DMEE elicits an anti-inflammatory effect by inhibiting the JNK/ERK/NF-κB signaling pathway in LPS-stimulated RAW 264.7 macrophages and has an anti-septic effect by blocking IL-6, which was found in the plasma of LPS-stimulated septic shock mice. Because of the anti-inflammatory properties of DMEE, it could be an effective therapeutic strategy in systemic inflammatory response syndromes such as septic shock or COVID-19.

## Figures and Tables

**Table 1:** Primer sequences used in the RT-PCR analyses.

Target Gene	Primer Sequence	
*iNOS*	F	5′-CATGCTACTGGAGGTGGGTG-3′
	R	5′-CATTGATCTCCGTGACAGCC-3′
*COX-2*	F	5′-TGCTGTACAAGCAGTGGCAA-3
	R	5′-GCAGCCATTTCCTTCTCTCC-3′
*IL-6*	F	5′-GAGGATACCACTCCCAACAGACC-3′
	R	5′-AAGTGCATCATCGTTGTTCATACA-3′
*IL-1β*	F	5′-ACCTGCTGGTGTGTGACGTT-3′
	R	5′-TCGTTGCTTGGTTCTCCTTG-3′
*β-actin*	F	5′-ATCACTATTGGCAACGAGCG-3′
	R	5′-TCAGCAATGCCTGGGTACAT-3′

## Data Availability

Data is contained within the article.
